# Acute Transient Sialadenitis After a Laparoscopic Cholecystectomy

**DOI:** 10.7759/cureus.67858

**Published:** 2024-08-26

**Authors:** Camila S Ríos de Choudens, Ana L Melero-Pardo, Cesar Luque-Fontanez

**Affiliations:** 1 Otolaryngology-Head and Neck Surgery, University of Puerto Rico School of Medicine, San Juan, PRI; 2 Medicine, Universidad Central del Caribe, San Juan, PRI; 3 Surgery, University of Puerto Rico School of Medicine, San Juan, PRI

**Keywords:** general anesthesia, anesthesia mumps, laporoscopic cholecystectomy, acute parotitis, sialadenitis

## Abstract

Transient acute sialadenitis after anesthesia, also known as "anesthesia mumps," is a rare phenomenon reported after surgery, typically associated with extensive surgeries. It is a complication that is usually self-resolving but, in rare cases, may lead to airway obstruction. The most common associated causes include dehydration, components of anesthesia, duct obstruction due to positioning, and external compression, among others. Here, we present the case of bilateral parotitis after an elective laparoscopic cholecystectomy in a 76-year-old male.

## Introduction

"Anesthesia mumps" is a rare complication of general anesthesia. It is characterized by transient inflammation and swelling of the salivary glands, specifically the parotid glands. This condition can be unilateral or bilateral, with reported incidence ranging from 0.2% to 17% [1, 2]. It is usually benign and can develop during or after surgery, typically resolving without sequelae within a few days. In rare cases, respiratory distress may occur, necessitating immediate intervention to secure the airway [2, 3]. Some predisposing factors for developing transient sialadenopathies include belladonna drugs, succinylcholine, and neuromuscular blocking agents, among others [4]. Certain reports attribute the condition to air entering the parotid glands retrogradely, causing pneumoparotitis, or to obstruction of the gland due to stress caused by continuous positive pressure or physical obstruction during prolonged procedures [1]. More commonly, however, it is attributed to drugs causing transient obstruction in patients who had a degree of dehydration by thickening salivary secretions [1]. Anesthesia mumps has been reported after various surgical procedures, including those in obstetrics and gynecology, vascular surgery, orthopedic surgery, and neurosurgery, typically lasting two or more hours.

## Case presentation

A 76-year-old male underwent a laparoscopic cholecystectomy due to a history of symptomatic cholelithiasis. The surgery was unremarkable, lasting one hour. The anesthesia team used midazolam, glycopyrrolate, fentanyl, dexamethasone, propofol, and rocuronium/sugammadex throughout the procedure. The patient was extubated after one hour and thirty minutes of anesthesia time without difficulty.

**Figure 1:**
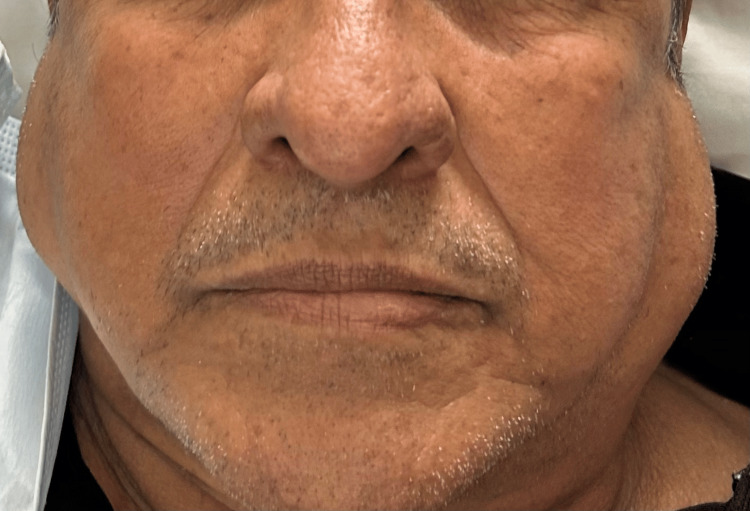
Patient with bilateral facial swelling evaluated on POD1. POD1: postoperative day 1.

**Figure 2:**
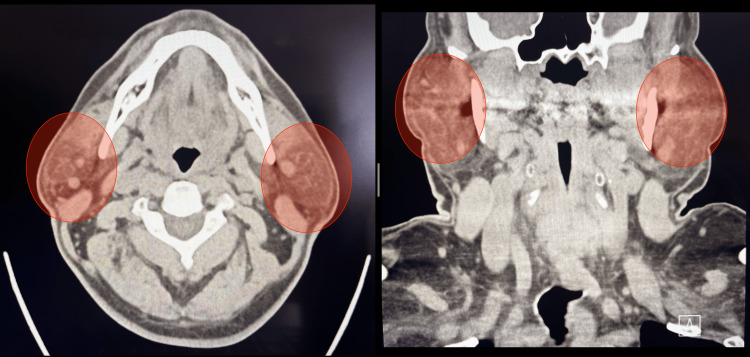
Axial and coronal planes of CT scan showing bilateral parotid swelling (highlighted in red).

During his immediate postoperative period, no complications were reported, and the patient was discharged home within hours of the procedure. On postoperative day 1, the patient returned to the emergency department due to bilateral facial swelling (Figure 1). The patient was in no acute distress, and no signs of respiratory distress were present. Physical examination was notable only for bilateral parotid gland swelling with mild tenderness to palpation. No secretions or suppuration were evident via Stensen’s duct. The patient was diagnosed with bilateral parotitis. A neck CT without contrast was performed, which revealed bilateral parotid swelling and free air around the glottis (Figure 2). The patient was provided with IV fluids, received one dose of IV antibiotics, and was discharged home. The patient was re-evaluated and showed complete resolution of symptoms by postoperative day 4.

## Discussion

Acute postoperative sialadenitis is a noninfectious process with an unknown etiology. Several hypotheses have been proposed to explain the pathophysiology behind the development of anesthesia mumps. One hypothesis suggests that head hyperextension or a degree of pneumoparotid due to increased positive pressure within the oral cavity may play a role. This increase in positive pressure may be caused by coughing or sneezing during the anesthesia or post-anesthesia period. In addition, the perioperative use of barbiturates, anticholinergics, beta-blockers, antihistamines, and phenothiazines increases the risk of salivary stasis. These medications may lead to the loss of muscle tone around Stensen’s duct via autonomic nerve stimulation, facilitating the retrograde passage of air into the parotid gland [4]. Another hypothesis is based on the activation of a pharyngeal reflex, which is triggered by straining or coughing against the endotracheal tube, leading to parasympathetic stimulation. This parasympathetic stimulation causes hyperemia in the parotid glands [4, 5]. Lastly, neck extension and tongue compression by the endotracheal tube can lead to salivary stasis via occlusion of the ducts [5]. The most commonly accepted theory suggests that patients with a degree of dehydration due to fasting may experience thickened saliva, leading to duct obstruction. In our case, the most likely cause was multifactorial, involving dehydration and anesthesia components. Dehydration may have caused crystallization of saliva, leading to a transient blockage of Stensen’s duct. In addition, the anesthesia components included glycopyrrolate, an anticholinergic drug that inhibits salivary gland secretions, and rocuronium, which also competes for cholinergic receptors. These risk factors likely contributed to the development of acute transient swelling of both parotid glands.

Postoperative sialadenitis is primarily a self-limited benign disease that resolves within hours to days. However, treatment typically includes rehydration and anti-inflammatory medications, such as corticosteroids or nonsteroidal anti-inflammatory drugs [6]. In this case, the patient was treated with rehydration and one dose of IV antibiotics. No further management was warranted, and resolution was observed within three days of onset.

## Conclusions

In brief, acute swelling of the parotid gland after anesthesia is a very rare occurrence. Physicians should be familiar with this complication to manage it accordingly. It should be noted that, while it is typically a transient and benign anesthesia-related complication, physicians must be aware that it may occasionally lead to urgent cases requiring immediate intervention to secure the airway. Based on our case, we concur with previous reports that anesthesia components and dehydration may contribute to the development of anesthesia mumps. To prevent this complication, we suggest avoiding direct compression of the ducts, minimizing neck manipulation to allow normal circulation (especially if the surgery is prolonged or the patient is placed in the prone position), promoting smooth intubation and extubation without significant straining, and maintaining optimal hydration status throughout the procedure.
